# SNAKE: A modular realistic fMRI data simulator from the space-time domain to k-space and back

**DOI:** 10.1162/IMAG.a.121

**Published:** 2025-09-02

**Authors:** Pierre-Antoine Comby, Alexandre Vignaud, Philippe Ciuciu

**Affiliations:** CEA, Joliot, NeuroSpin, Université Paris-Saclay, Gif-sur-Yvette, France; Inria, MIND, Université Paris-Saclay, Palaiseau, France

**Keywords:** fMRI, brain imaging, accelerated sampling, compressed sensing, simulation, open source, python

## Abstract

We propose a new, modular, open-source, Python-based 3D+time realistic functional magnetic resonance imaging (fMRI) data simulation software. SNAKE or *S*imulator from *N*eurovascular coupling to *A*cquisition of *K*-space data for *E*xploration of fMRI acquisition techniques. It is the first simulator to simulate the entire chain of fMRI data acquisition, from the spatio-temporal design of evoked brain responses to various 3D sampling strategies of k-space data with multiple coils. We now have the possibility to extend the forward acquisition model to different noise and artifact sources while remaining memory-efficient. Using this in-silico setup, we can provide a realistic and reproducible ground truth for fMRI reconstruction methods in 3D accelerated acquisition settings and explore the influence of critical parameters. This includes the acceleration factor and signal-to-noise ratio (SNR), on downstream tasks of image reconstruction and statistical analysis of evoked brain activity. In this paper, we present three scenarios of increasing complexity to showcase the flexibility, versatility, and fidelity of SNAKE: From a temporally fixed full 3D Cartesian to various 3D non-Cartesian sampling patterns, we can compare—with reproducibility guarantees—how experimental paradigms, acquisition strategies, and reconstruction methods contribute and interact together, affecting the downstream statistical analysis.

## 1 Context and Motivation

Functional Magnetic Resonance Imaging (fMRI) has emerged as a powerful tool in neuroscience, allowing scientists to investigate human brain function non-invasively. By measuring $T_{2}^{*}$ variations over time due to changes in blood oxygenation and flow, the BOLD effect (Ogawa et al., 1990), induced either in response to external stimuli or spontaneously at rest, has provided invaluable insights into cognitive processes, intrinsic functional networks, and brain pathology. However, conducting task-based fMRI experiments is an expensive and time-consuming endeavor, often requiring access to advanced imaging facilities and substantial expertise in protocol design, data collection, and analysis. In addition, the reproducibility of fMRI experiments and findings has been shown to be a critical issue in cognitive and clinical neuroscience (Bossier et al., 2020; Griffanti et al., 2016; Liou et al., 2006; Nee, 2019).

However, a major roadblock to replicating fMRI studies is the sample size, as most studies involve small cohorts and are less replicable mainly due to multiple sources of variability (between subjects, MR systems, etc.; Marek et al., 2022; Nakuci et al., 2023). In addition, accommodation and habituation effects limit the repeatability of within-subject tests (Bennett & Miller, 2010), severely hindering the comparison of acquisition protocols.

This variability prevents the development of new methodologies to address the growing needs in the fMRI literature, particularly the quest for higher temporal (Viessmann & Polimeni, 2021) and spatial (Pinho et al., 2020) resolution. The development of complex methods on data acquisition and image reconstruction in (f)MRI faces challenges in providing a fair comparison due to limited existing ground-truth data collected on the same individuals under different imaging setups. Recent advances in computing power have led to the emergence of (f)MRI simulators that aim to address this limitation (see Welvaert & Rosseel, 2014 for a review). However, most current simulators are limited to producing magnitude-only (f)MRI images and lack raw k-space data, severely hindering their usability for validation of acquisition and reconstruction methods. On the other hand, anatomical MRI simulators typically rely on Bloch’s model (Castillo-Passi et al., 2023), but are too computationally demanding and lack parametrization for the fMRI experiment.

In search of absolute ground truth, other solutions in addition to simulation have been proposed, such as active phantoms (Cheng et al., 2006; Kumar et al., 2021), fMRI monitoring with other devices (Caballero-Gaudes & Reynolds, 2017), or removal of acquisition artifacts (Amor, Le Ster, et al., 2024; Bollmann et al., 2017; Duerst et al., 2015). More recently, the development of generative Artificial Intelligence (AI) has opened new opportunities for the synthesis of realistic MRI data (Gopinath et al., 2024; Pinaya et al., 2023). However, it requires expensive computation that would not scale for generating high-resolution 3D+time fMRI data, and its lack of explicability over the yielded sequence of fMRI volumes discards it for validating image reconstruction algorithms and statistical analysis.

In general, none of the proposed solutions has succeeded in providing a (i) a full control on both the temporally resolved input hemodynamic signal and the output BOLD fMRI time series, (ii) an easy-to-use and reproducible framework through open-source software, and (iii) a low computational or operational cost.

*This paper aims to fill this gap. More precisely, our contribution can be summarized as follows:*

We propose a realistic fMRI simulator, named SNAKE based on an *extended* Fourier model of MRI data acquisition that can create all the required k-space data for evaluating the fMRI processing tool chain: From the definition of an experimental paradigm and the localization of brain activation in a realistic phantom, up to the generation of 3D+time k-space data. This bottom-up approach gives fMRI scientists a level playing field to explore and compare various properties of acquisition and reconstruction strategies, such as the choice of the sampling pattern (Cartesian vs. non-Cartesian readouts), the acceleration factor, the signal-to-noise ratio (SNR), etc. SNAKE could as well be used in the near future to train deep learning models for fMRI image reconstruction, by providing fully parameterized high-quality reference data.

To compare the reconstruction methods in more challenging settings, the ground-truth data can be degraded in various ways before and during the acquisition process (motion, static field inhomogeneities, thermal and physiological noise, etc.).

The simulator primarily produces a sequence of k-space volumes sampled with realistic fMRI sequences. Furthermore, for completeness, we offer the possibility to produce a sequence of fMRI volumes in the image space by plugging in simple but efficient *volume-wise* reconstruction algorithms. SNAKE has been designed to be as light and as fast as possible to give its users an upper limit to the statistical results that an fMRI experiment can achieve, given the acquisition and reconstruction strategies chosen.

In the remainder of this paper, we first propose a review of the available fMRI simulation tools, highlighting their strengths and weaknesses (Section 2). Then, we present the underlying model (Section 3.1) and the design of SNAKE (Section 4). Finally, we present three typical simulation scenarios (Section 5), with basic image reconstruction and statistical analysis (Section 6), to illustrate the various possibilities of SNAKE, and discuss further the strength and limitations of Section 7.

In addition to this paper, we also provide a detailed documentation and a set of tutorials to help users get started with SNAKE in the accompanying documentation available at https://mind-inria.github.io/snake-fmri.

## 2 Existing Software and Their Limitations

The intricacies of fMRI data create significant obstacles in developing a unified framework for generating comparable datasets. A detailed examination of the existing literature on fMRI simulation, as highlighted by Welvaert and Rosseel (2014), has shown that the lack of a standardized approach to synthesizing fMRI data severely hinders reproducibility in fMRI research. This underscores the need for improved transparency in the reporting of experimental design and a more nuanced understanding of the processes involved in the acquisition of fMRI data. In addition, there exist anatomical MRI simulators based on the Bloch equations that generate contrast-weighted MR images, but they usually remain confined to anatomical imaging with no straightforward extension to fMRI. A detailed comparison of the currently available MRI simulators is provided in Castillo-Passi et al. (2023).

In what follows, we provide a broad overview of available MRI and fMRI simulators in Table 1 and focus hereafter on the main alternative to SNAKE, in increasing order of similarity.

**Table 1.** Summary of characteristics of different fMRI data simulators.

	Simulator name	Source	API	Sim. domain	External data	Interface	Reconstr.	Ecosystem
	The Virtual Brain Sanz Leon et al., 2013	GPL-3.0	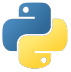	Image		GUI/script	N/A	N/A
**MRI**	Jemris Stöcker et al., 2010	GPL-2.0	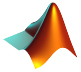 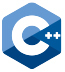	Bloch		GUI	ISMRMD raw data	N/A
	ODIN Jochimsen et al., 2006	GPL-2.0	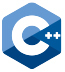	Bloch	Tissue Maps, Sequence	c++/GUI	FFT	OdinReco
	MRILab Liu et al., 2017	BSD-2	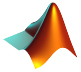 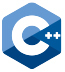	Bloch	Preset Macros	GUI	FFTNon-Cartesian	GUIGadgetron
	Bloch-Solver Kose and Kose, 2017	Proprietary	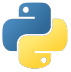	Bloch	Tissue Maps,	script	FFT	N/A
	Fabian Lajous et al., 2022	BSD-3	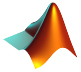	Bloch (EPG)	Tissue Maps, Sequence	script	FFT	N/A
	KomaMRI Castillo-Passi et al., 2023	MIT	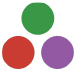	Bloch	Pulseq	GUI	FFT	Pulseq
**fMRI **	POSSUM Drobnjak et al., 2006	FSL	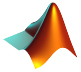 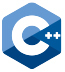	Bloch	Tissue Maps Sequence, Events	CLI	FFT	FSL
	Neurolib Cakan et al., 2023	MIT	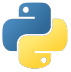	Image	Connectivity Matrices	script	N/A	Jupyter
	SimTB Erhardt et al., 2012	Open Source	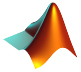	Image	Spatial Maps, Events	GUI	N/A	MATLAB
	NeuRoSim Welvaert et al., 2011	GPL-2.0	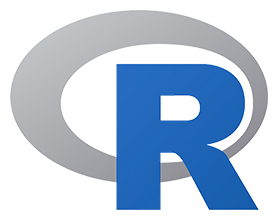	Image		script	N/A	N/A
	*fmriSim* *Ellis* et al., 2020	*Apache-2.0*	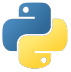	*Image*		*script*	*N/A*	*Brainiak*
	SNAKE-fMRI	MIT	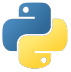	Kspace Image	Configuration files	script/CLI	Any (4D methods)	Any

API Languages: 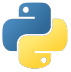
 Python; 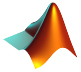
 MATLAB®; 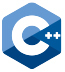
 C++; 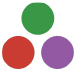 Julia; 
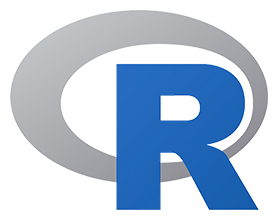
 R

Among existing fMRI data simulation tools, several offer distinct advantages and limitations. The Virtual Brain (Sanz Leon et al., 2013; Schirner et al., 2022) is an open-source multi-modal brain simulator, focusing on the simulation of brain activity using the structural connectome and interaction between functional networks. It is capable of yielding voxel-wise BOLD fMRI time series. However, it remains complex to master and is not a dedicated tool for analyzing the acquisition/image reconstruction chain in fMRI.

fMRIsim, proposed by Ellis et al. (2020), is a Python package that enables standardized and realistic fMRI time series simulation in the image domain. It was inspired by a parent R package neuRosim (Welvaert et al., 2011). It allows for the evaluation of complex experimental designs and optimization of the statistical power. However, it focuses on single-subject simulations and requires manual parameter setting or estimation from real data. Moreover, it primarily deals with magnitude data and uses additive noise settings, which might restrict its applicability to specific simulations or use cases (e.g., limited resolution).

SimTB, introduced by Erhardt et al. (2012), is a MATLAB toolbox specialized in simulating fMRI time series using a separable spatio-temporal model. It offers extensive customization options, including spatial sources, experimental paradigms, tissue-specific properties, noise, and head movement. SimTB is equipped with both a graphical user interface and scripting capabilities. However, no k-space data are available to assess the performance of various image reconstruction algorithms.

The POSSUM simulator, as outlined by Drobnjak et al. (2006), offers a comprehensive approach to the impact of specific artifacts encountered in the acquisition of fMRI data. POSSUM accurately simulates these artifacts using Bloch equations and a geometric definition of the brain. However, its computational cost is high, making simulations of full brains at high resolution prohibitive. Additionally, it currently only offers the possibility to yield Cartesian data in k-space. FMRI analysis is deferred to the FSL library, and no comparison with the ground truth is provided as an outcome of the toolbox.

## 3 Extended Fourier Model for fMRI Acquisition

To bridge the gap between image and k-space simulation, a more realistic forward model is needed. Using the Bloch equation model (Bloch, 1946) would be prohibitively expensive for whole-brain simulation at high spatial and temporal resolution; instead, we extend the classical model based on the Fourier transform.

### 3.1 Single-shot acquisition

Considering the multicoil imaging setup, $T_{2}^{*}$ relaxation and off-resonance effects due respectively to signal decay and $B_{0}$ inhomogeneities over the field of view (FOV), the signal acquired by the MR system for the s-th shot in the ℓ-th coil is defined as follows:

$$y_{\ell ,s}\left(t\right)=\int_{FOV}m(r,t)S_{\ell }(r)e^{-2\iota \pi \Delta f_{r}\left(r\right)t}e^{-2\iota \pi k_{s}\left(t\right)\;\cdot r}dr$$ (1)

where $k_{s}$ is the k-space trajectory for the s-shot with $k_{s}(t)$ the k-space location at time t, $\Delta f_{r}(r)$ the static $B_{0}$ inhomogeneity map, $S_{\ell }(r)$ the sensitivity map for coil ℓ∈{1,…,L} , m(r, t) the base magnetization of the image. Note that Eq. (1) holds for both 2D and 3D imaging. However, the main focus of this paper will be 3D acquisitions, which can proceed to segment the readout either across planes for stacked strategies (e.g., 3D EPI, stack of spirals) (Poser et al., 2010) or across shots for full 3D non-Cartesian strategies (e.g., SPARKLING (Amor, Ciuciu, et al., 2024) or TURBINE (Graedel et al., 2022)). In the former case, we will use $k_{s}(t)=[k_{s}^{x}(t),\;k_{s}^{y}(t)]$ at a given elevation z-th embedded in the definition of s, whereas in the latter case we will consider $k_{s}(t)=[k_{s}^{x}(t),\;k_{s}^{y}(t),\;k_{s}^{z}(t)]$ .

**Appendix Fig. A1.**
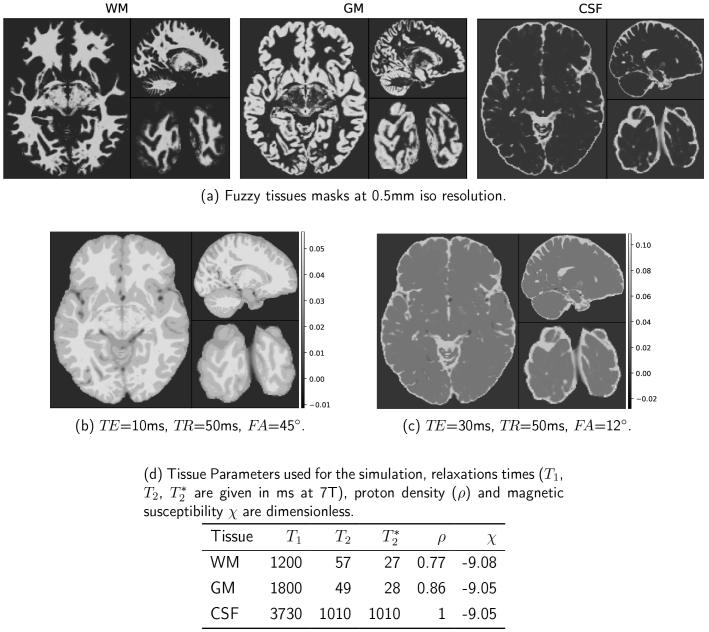
BrainWeb phantom (Aubert-Broche et al., 2006) and tissue parameters used for the simulations.

Assuming steady-state and perfect spoiling regimes, the signal obtained from a Gradient Recall Echo (GRE) sequence parameterized by (TR, TE, α) , with $T_{1}\gg TR$ , is:

$$m(r,\;t)=\underset{\mu (r,t_{ref})}{\underset{︸}{\rho (r)\text{sin}\alpha \frac{1-e^{-TR/T_{1}(r)}}{1-\text{cos}\alpha e^{-TR/T_{1}(r)}}e^{-t_{ref}/T_{2}^{*}(r)}}}e^{\left(t_{ref}-t)/T_{2}^{*}(r\right)}$$ (2)

where $\rho (r),T_{1}(r)$ , and $T_{2}^{*}(r)$ are quantitative maps of the proton density, longitudinal ($T_{1}$), and transverse ($T_{2}^{*}$) relaxation parameters, respectively, and have fixed values during the shot. Different contrasts can be generated, such as that shown in Appendix Figure A1. $\mu (r,\;t_{ref})$ describes the state of the object (i.e., its contrast) obtained after the excitation pulse at a point of reference with respect to relaxation $t_{ref}$ , in the case of the in-out sampling pattern, we typically have $t_{ref}=TE$ , the echo time centered on the $T_{obs}$ long read-out window with $t\in \left[-\frac{T_{obs}}{2},\frac{T_{obs}}{2}\right]$.

To simplify the calculation, the quantitative maps are separated into $N_{tis}$ tissue types, each with a set of constant MR parameters $\rho T_{1}$ and $T_{2}^{*}$:,

$$m(r,\;t)=\sum_{i=1}^{N_{tis}}m_{i}(r,\;t)=\sum_{i=1}^{N_{tis}}w_{i}(r)\;\mu_{i}(t_{ref})e^{(t_{ref}-t)/T_{2,i}^{*}}$$ (3)

where $w_{i}$ is the proportion of tissue i in the voxel location r, $\mu_{i}(t_{ref})$ (or $\mu_{i}$ for brevity) the contrast of the i-th tissue at time $t_{ref}$ (which depends the tissue’s $T_{1,i}$ ) and $T_{2,i}^{*}$ the $T_{2}^{*}$ relaxation parameter of the i-th tissue type.

Combining Eqs. (1) to (3), we obtain the complete signal equation for a single coil ℓ and a single shot s:

$$\begin{array}{cc} y_{\ell ,s}\left(t\right) & =\int_{FOV}\overset{N_{tis}}{\underset{i=1}{\sum \;}}w_{i}(\text{r})\;\mu_{i}e^{-t/T_{2,i}^{*}}\;S_{\ell }(\text{r})e^{-2\iota \pi \Delta f_{r}(\text{r})t}e^{-2\iota \pi k_{s}\left(t\right)\;\cdot \text{r}}d\text{r}\\ & =\overset{N_{tis}}{\underset{i=1}{\sum \;}}\mu_{i}e^{-t/T_{2,i}^{*}}\int_{FOV}w_{i}(\text{r})\;\;S_{\ell }(\text{r})e^{-2\iota \pi \Delta f_{r}(\text{r})t}e^{-2\iota \pi k_{s}\left(t\right)\;\cdot \text{r}}d\text{r}. \end{array}$$ (4)

The static off-resonance term $e^{-2\iota \pi \Delta f_{r}(\text{r})t}$ can be approximated by a sum of separable bilinear terms $\sum_{p=1}^{P}c_{p}\left(t\right)b_{p}\left(r\right)≃e^{-2\iota \pi \Delta f_{r}\left(\text{r}\right)t}$ (Amor et al., 2023; Sutton et al., 2003), where P is the number of interpolator used to approximate the decomposition.

Furthermore, considering the set of k-space sampling points ${\text{k}}_{s}t_{1},...,{\text{k}}_{s}t_{n},...,{\text{k}}_{s}t_{N}$ that are acquired possibly off the Cartesian grid at times $t_{n}=(n-1)\Delta t$ (where Δt is typically the dwell time of the scanner, in the order of 10 μ**s**), and the spatial locations ${\left(r_{m}\right)}_{m=1}^{M}\in {\mathbb{N}}^{3\times M}$ in $M=N_{x}N_{y}N_{z}$ voxels to cover the FOV, we obtain:

 yℓ,s[tn]  =∑i=1Ntisμie−tn/T2,1∗∑p=1PCp[tn]∫FOVwi[r]Sℓ(r)bp(r)e−2ιπkS[tn] ⋅rdr                     =∑i=1Ntisμie−tn/T2,1∗ ∑p=1PCp[tn] ∑m=1Mwi[rm]Sℓ[rm]bp[rm]e−2ιπkS[tn] ⋅rm                     =∑i=1Ntisμie−tn/T2,1* ∑p=1PCp[tn] F{bpSℓwi}[kS[tn]]. (5)

The resulting signal is a doubly weighted sum of Fourier transforms (ℱ) first by interpolation coefficients in the k-space ($b_{p}$) and second by tissue-specific contrast and $T_{2}^{*}$ decay: $\mu_{i}e^{-t_{n}/T_{2,i}^{*}}$. In general, to generate the k-space data $y=(y_{1},\ldots ,y_{L})$ in the multicoil array, for a single shot s, the total number of Fourier transform calls in Eq. (5) is $N_{tis}\cdot P\cdot L$ . The computational cost may be reduced by limiting the number of tissues to a few (e.g., 3 like gray matter, white matter, and cerebrospinal fluid) and/or neglecting $T_{2}^{*}$ relaxation (which is admissible if $T_{obs}=N\Delta T\ll T_{2}^{*}$) and off-resonance effects. Making all these hypotheses leads to the following *Basic Fourier* model:

$$y_{\ell ,s}[t_{n}]=ℱ\left\{S_{\ell }\sum_{i=1}^{N_{tis}}\mu_{i}w_{i}\right\}[{\text{k}}_{s}[t_{n}]]=ℱ\left\{S_{\ell }\mu \right\}[{\text{k}}_{s}[t_{n}]].$$ (6)

Note that Eq. (6) is the model used as the basis for the reconstruction algorithms. However, neglecting $T_{2}^{*}$ relaxation in fMRI acquisition may yield misleading results, in particular for long-readout trajectories (such as EVI) or if two neighboring points in the k-space are sampled at the two extremities of the sampling trajectory.

### 3.2 BOLD as a TE-sensitive change between shots

**Fig. 1.**
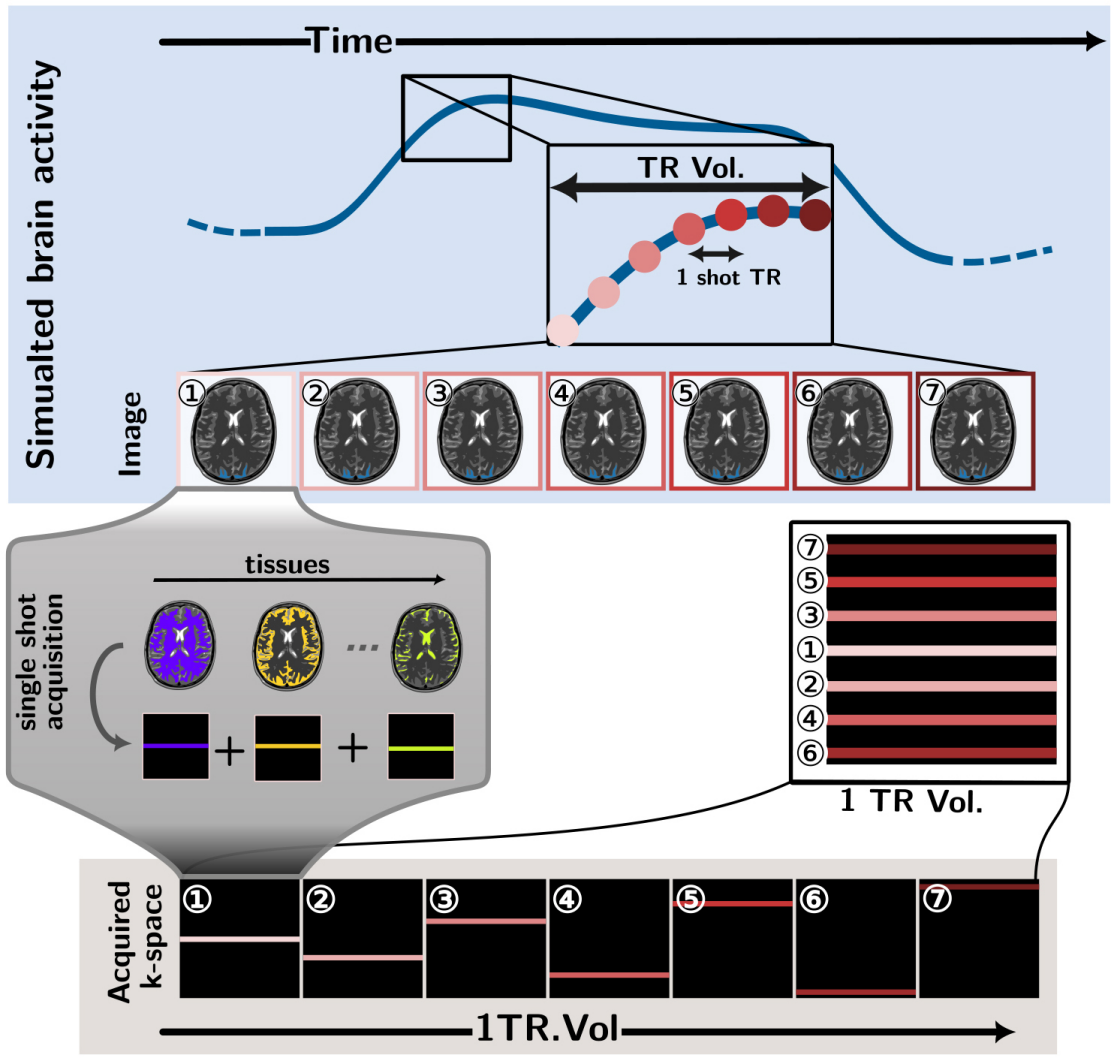
Acquisition method implemented in SNAKE—The case represented is simplified to a 2D Cartesian case (e.g., a projected view of a 3D non-accelerated EPI scheme). Each shot (i.e., a plane in 3D EPI) of the k-space sampling pattern is acquired separately from an on-the-fly simulated volume in the image domain as shown in the blue frame. The shots are numbered here from ① to ⑦. The parallel acquisition is performed in parallel for each tissue type to apply the $T_{2}^{*}$ relaxation model (5).

The previous section introduced the acquisition model for a single shot. In practice, acquisition consists of multiple shots, acquired at every $TR_{shot}$ . In the context of 3D fMRI, these shots are then grouped together to build a single k-space volume (see Fig. 1). Adding fMRI capability, that is, sensitivity to the BOLD effect, is achieved by modifying simulation parameters between consecutive shots. In particular, the BOLD effect is modeled as a change of $T_{2}^{*}$ following brain activity. If we consider that the BOLD effect modifies the baseline gray matter $T_{2}^{*}$—in a given region of interest, and in a simplified manner—as $T_{2,BOLD}^{*}=\frac{1}{R_{2,GM}^{*}+\Delta R_{2}^{*}}$ (Jin et al., 2006; Uludağ & Kasper, 2023), the contrast in that region is updated as follows:

$$\begin{array}{c} \begin{array}{c} \mu_{BOLD\;=\;S_{0}\;\cdot \;\text{exp}(-\text{TE}/T_{2,BOLD}^{*})=\;S_{0\;}\cdot \;\text{exp }(-TE\;\cdot \;(R_{2,GM}^{*}+\;\Delta R_{2}^{*}))}\\ \mu_{BOLD\;=\;\mu_{GM}\text{exp}(-TE\;\cdot \;\Delta R_{2}^{*})\;≃\;\mu_{GM}(1-TE\;\cdot \;\Delta R_{2}^{*}).} \end{array} \end{array}$$ (7)

where $S_{0}$ denotes the net magnetization. Hence, for every shot s, we can determine the base intensity, in the image domain:

$$\mu_{BOLD}(t_{s})=(1-TE\cdot \Delta R_{2}^{*}\tilde{h}(t_{s}))\;\cdot \mu_{GM}(t_{s}),$$ (8)

where $\tilde{h}(t)$ is the normalized hemodynamic response such that $\text{max}\left\{\tilde{h}\left(t\right)\right\}=1$ . In the context of task-based fMRI, it is modeled by convolving a sequence of events (event-related paradigms) or blocks (block paradigms) with a reference hemodynamic response function HRF (Ciuciu et al., 2003; Glover, 1999). In our simulation, we used $\Delta R_{2}^{*}=-1$ Hz (following the value used by Jin et al., 2006), which generates a 2.5% increase in the BOLD contrast at TE=25 ms.

### 3.3 Noise and SNR calibration

The (f)MRI signal in the k-space is also corrupted by thermal noise sources that arise from the acquisition process in two forms: Brownian motion of spins and random fluctuation in the RF receiver processing chain.

To model those effects, we add a complex multivariate Gaussian noise over the coil component ℓ∈(1,…,L) for every shot s. An existing coil covariance matrix $\Sigma \in {\mathbb{C}}^{L\times L}$ can be supplied to match an existing hardware set-up. Moreover, the global noise variance can be tuned manually, to set the *input SNR* in k-space. Concretely, the noise for each time point in the shot is sampled from ${\left(n_{\ell ,s}\left[t_{n}\right]\right)}_{1\leq \ell \leq L}∼N\left(0,\frac{E\left(\hat{x}\right)}{SNRi}\Sigma \right)$ where Σ is the coil covariance matrix. $E(\hat{x})$ is the energy of the *ideal* phantom, acquired at TE , and $SNR_{i}$ is the input SNR defined by the user in k-space. To add noise to a full shot, we draw N realizations of the L-dimensional noise vector ${\left(n_{\ell ,s}\left[t_{n}\right]\right)}_{\begin{array}{c} 1\leq l\leq L\\ 1\leq n\leq N \end{array}}$ . Then, noisy data are formed as follows:

$$\forall t_{n}\in 1,\ldots ,N,\;\;\;\;\;{\tilde{y}}_{\ell ,s}\left[t_{n}\right]=y_{\ell ,s}\left[t_{n}\right]+n_{\ell ,s}\left[t_{n}\right].$$ (9)

It is possible to calibrate the value of SNRi using experimental data, by computing the energy ratio of k-space shots collected with and without RF excitation.

Another solution is to view the joint system of acquisition and reconstruction together, and consider a known case of image quality output. In this case, the value of $SNR_{i}$ is tuned to obtain the desired image quality, gathering all missing modeling aspects in this Gaussian noise.

More structured noise sources (e.g., physiological noise such as heartbeat, breathing rate, motion) could be superimposed on the simulated signal. However, the wide variety of options in this field for modeling such noise components is beyond the scope of this article. However, SNAKE already offers a wide flexibility through the handler mechanism (see Section 4.1). Future work (open to contributions) will go into more detail on the implementation of these noise sources.

#### 3.3.1 Extending the signal model

**Fig. 2.**
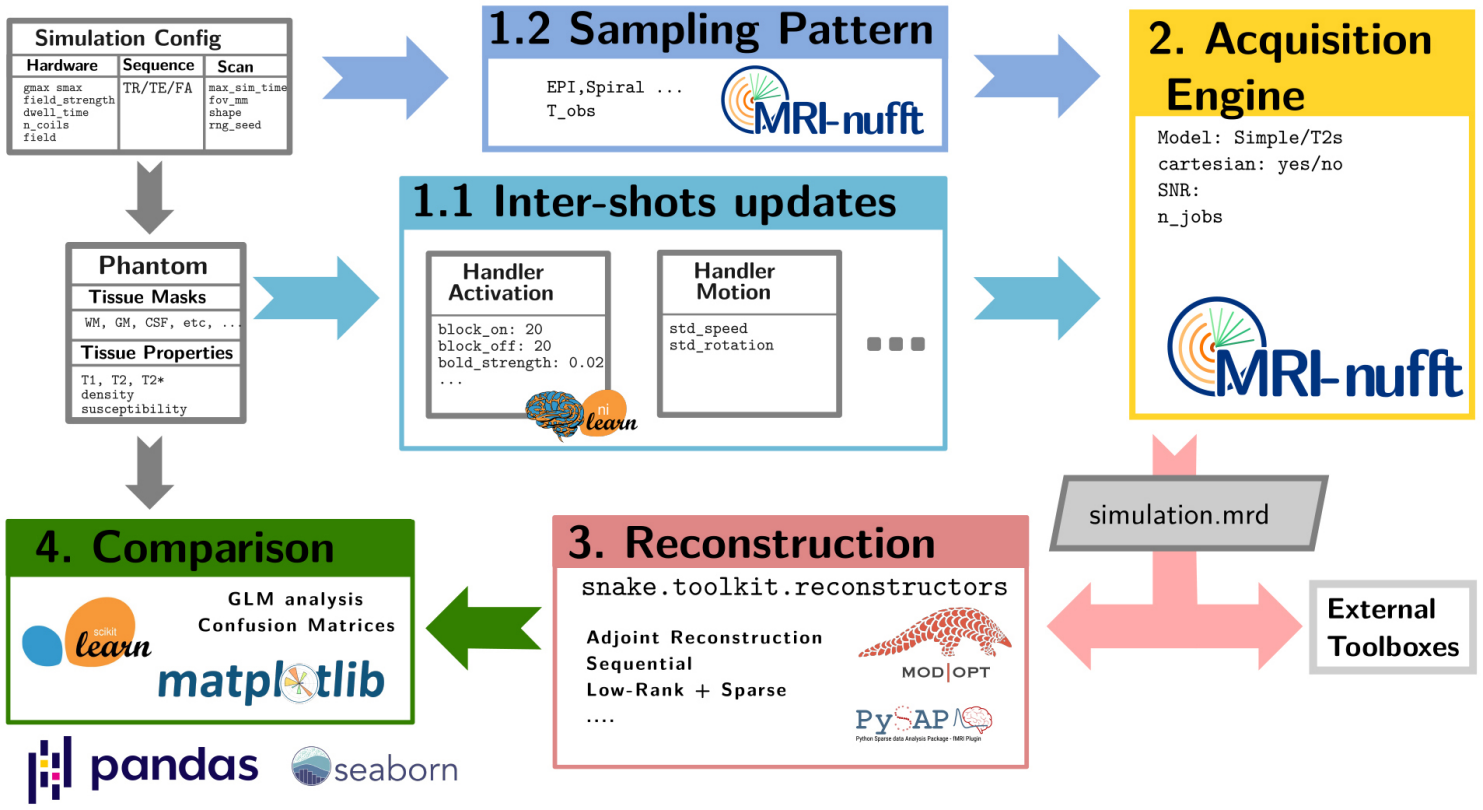
Modular design of the SNAKE simulator, which embeds an ecosystem of Python packages for its different building blocks. 1. The signal variations in the brain (BOLD, motion, etc.,…) are simulated, along with the sampling pattern for each shot. 2. The acquisition engine gathers the multiple shots involved in the acquisition as well as the current state of the 3D anatomical phantom. It relies on the MRI-nufft package for non-Cartesian acquisition. 3. Then, the output k-space data are reconstructed into the image domain using some specific method implemented in pysap-fmri/ModOpt (Farrens et al., 2020), thereby producing an estimation of the fMRI time-series. 4. Finally, the sequence of fMRI volumes is analyzed through a general linear model (GLM) defined from the experimental paradigm and implemented in nilearn (Abraham et al., 2014). Statistical maps are produced and compared to the ground truth used at the simulation stage using confusion matrices and statistical metrics (accuracy or ACC, balanced accuracy or BACC to correct for imbalanced dataset in fMRI).

In Section 3.1, we presented the acquisition model of SNAKE. In Section 3.2, we explain how the BOLD contrast is changed between the acquisition of two consecutive shots. Similarly, other modifications of the acquired data could occur between shots due to the presence of motion, physiological noise, or other perturbations, which can be modeled in the future using the handler mechanism (as described in Fig. 2). For example, we present the effect of motion as an example in the accompanying software documentation.^*^ Concretely, for the motion case, we would update the position of all tissue masks $w_{i}$ (i.e., with a temporal resolution of $TR_{shot}≃50\;\text{ms}$ ) and then proceed to acquire the next shot.

### 3.4 Summary of general hypotheses on the acquisition model

In general, the current capabilities of SNAKE are restricted by the following hypotheses.

(i) Tissue parameters and physiology are frozen during the acquisition of a single shot: ρ, $T_{1}$, $T_{2}^{*}$, χ are constant when the signal in Eq. (5) is computed.
(ii) The BOLD effect is linearly sensitive to TE .
(iii) The BOLD effect does not modify the phase of the signal.
(iv) Complex valued Gaussian noise is added in the k-space over all coil channels (modeling the thermal noise). The noise level is calculated from a user-prescribed input SNR.
(v) The acquisition of shots follows Eq. (5). As Section 6 will demonstrate, this model can be simplified to Eq. (6) if the trajectories do not have long readouts and are temporally smooth.

These are the minimal hypotheses on which the implementation of SNAKE is based. However, it also provides a flexible framework for adding more complex models (e.g., motion or other physiological perturbations) through the handler mechanism as described in Section 4.1. To make SNAKE user-friendly and reduce its parameterization, additional hypotheses can be formulated. However, the potential bias introduced by these simplifications should be carefully considered. In Section 5.1.1, we describe further restricting hypotheses for our study case.

It should be noted that SNAKE does not aim to reproduce the full complexity of MR physics, but rather to produce a realistic and reproducible framework that allows us to simulate various acquisition scenarios in fMRI with full control over the ground truth and its parameterization. As such, SNAKE provides an upper bound on the statistical sensitivity/specificity compromise given the definition of an acquisition and image reconstruction set-up. Hence, SNAKE is defined as an instrumental framework for benchmarking fMRI reconstruction methods that comes with statistical analysis tools to perform end-to-end validation of fMRI acquisition and reconstruction methods, as shown in Figure 2.

## 4 Main Characteristics of Snake Implementation

SNAKE has been designed as a fully reproducible modular fMRI simulator capable of generating k-space data efficiently. In what follows, we give a broad overview of the main features of SNAKE. Then, these features are illustrated in Sections 5 and 6.

### 4.1 Modular approach

SNAKE adopts a modular approach to simulate 3D + time fMRI data.

Typically, the simulation begins with the definition of an anatomical phantom of the brain in the image domain (see Appendix Fig. A1 in Appendix A), and proceeds to add BOLD contrast and various noise sources through object called “handlers” before generating the k-space data from a user-defined sampling pattern. As shown in Figure 2, these handlers can be chained to produce complex behaviors from simple operations. Moreover, numerous sampling patterns (especially non-Cartesian ones) can be generated through the use of the MRI-NUFFT library. The acquisition process is depicted in Figure 1. SNAKE supports both models described in Eqs. (5) and (6), respectively, through dedicated “engines” that are optimized for parallel computing of shots on GPU.

This option allows users to assess the need for a more complex, possibly more computationally demanding model in the context of the chosen scenario.

Moreover, SNAKE provides direct access to variational reconstruction methods using PySAP-fMRI^†^ and ModOpt (Farrens et al., 2020).

At the end of the processing chain, we can compare the reconstructed fMRI images and the corresponding time series voxel-wise or region-wise with their respective simulated ground truth, and then evaluate the effect of acquisition parameters (SNR, acceleration factor, etc.) and reconstruction strategies (density compensated adjoint Fourier, compressed sensing reconstruction, etc.) on image quality, as well as on statistical sensitivity/specificity compromise. Statistical analysis is performed using the nilearn package (Abraham et al., 2014).

### 4.2 Performance, reproducibility, and scalability made easier for neuroscientists

#### 4.2.1 Performance

Storing in RAM the full high spatiotemporal simulation is challenging at the high spatial and temporal resolution (340 gigabytes are required for a 1 mm-iso volume, with a unitary TR of 50 ms shot-wise for 5 min of a typical fMRI run), and each substantial change, like adding noise, would create a new copy of these data. Instead, we propose to yield the data to be acquired shot-wise on the fly, as each time point in the time series can be computed from a sequence of transformations applied to a single anatomical volume (for instance, adding the BOLD contrast, noise, or using motion parameters). Moreover, whenever possible, the computations are performed on a GPU, and shot-wise acquisitions are performed in parallel; the data are also eagerly offloaded to a hard disk when it is no longer required for computations. The k-space data generated by SNAKE is exported in the standardized ISMRMRD format (.mrd; Inati et al., 2016).

#### 4.2.2 Reproducibility

Enabling reproducibility in the study of fMRI processing methods and their benchmarking is at the heart of the development of SNAKE. This simulator can be installed directly from the Python package archive (https://pypi.org/project/snake-fmri/) and its core only depends on standard and well-tested Python packages. The simulation setup can be shared via .yaml files that describe the recipe to build a simulated scenario.^§^

#### 4.2.3 Scalability and interoperatibility

Using the .mrd file as the output, we open the door for interoperability of SNAKE with other toolboxes for image reconstruction, such as SigPy (Ong & Lustig, 2019) or BART (Uecker et al., 2015). We also provide an optimized data loader for the .mrd files generated by SNAKE.

Furthermore, based on the hydra framework (Yadan, 2019) we can run multiple simulations with different parameters or handlers and perform image reconstruction of fMRI volumes and simple statistical data analysis to compare competing approaches. SNAKE also allows us to scale up simulations from a single laptop to high-performance computing clusters.

## 5 Numerical Experiments

In this section, taking advantage of the modularity and scalability of SNAKE, we demonstrate the use of a controlled simulation framework to explore the potential benefits and challenges of moving to higher resolution in space and time for fMRI experiments. The results and analysis are presented in the next section.

### 5.1 Acquisition scenarios

First, we detail three simulated scenarios of increasing complexity (from 3D Cartesian low spatial resolution to 3D non-Cartesian high temporal or spatial resolution) with basic reconstruction and statistical analysis pipeline. All scenarios simulate a 5-min run with full brain coverage during a simple visual stimulation using a standard block-design paradigm at 7T (in terms of SNR), which alternates 20 sec-on and 20 sec-off periods. In response to visual stimuli, we induce a 2.5% change in BOLD contrast (following Eq. (7)) in a region of interest (ROI) in the occipital cortex. This ROI is defined from a fuzzy segmentation of gray matter that intersects an ellipse located in the occipital cortex.

Furthermore, we ran both the $T_{2}^{*}$ relaxation model (cf. Eq. (5)) and the simplified Fourier model (cf. Eq. (6)) for data acquisition, to determine in which settings a more complicated model is required.

#### 5.1.1 Working hypotheses to simplify acquisition

In all scenarios, the acquisition model considers only the intrinsic phenomenon of MR physics and leaves aside all other modeling steps (motion, physiological noise, off-resonance effects) that could be compensated using a more complicated reconstruction setup. In particular, in addition to the fundamental hypothesis in Section 3.4, we assume that:

- Following Hypothesis (iv) the coil covariance matrix Σ is set to identity, and we use a user-defined input SNR level (SNRi=1,000 , determined using the calibration step described in Section 3.3).
- *There is neither motion nor physiological perturbation (aside from the BOLD signal)*: In the context of our benchmarking of acquisition and reconstruction methods, we assume that the motion could be compensated for, even though this task may require, in practice, navigator echoes or dedicated hardware.
- *Off-resonance effects are not considered*: From this point of view, we are in an ideal situation with minimal static and dynamic $\Delta B_{0}$ inhomogeneities. Furthermore, we recently showed that these dynamic inhomogeneities can be corrected using a field camera, in addition to a static field map $\Delta B_{0}$ in the forward model at the reconstruction time (cf. Amor, Le Ster, et al., 2024; Amor et al., 2023) Eq. (5).
- *Reducing the number of tissues*: Limiting ourselves to the three cortical tissues (white matter (WM), gray matter (GM), cerebrospinal fluid (CSF)) does not hinder the comparison between ground-truth and reconstructed images or statistical analysis.

Such assumptions allow us to focus on the core components of SNAKE (see Section 3.4), reduce the computational load, but, nevertheless, provide an upper bound on the performance of the tested scenarios. Possible extensions can be added by implementing the appropriate handlers.

The three scenarios and their computational cost are summarized in Table 2. Details of all parameters used are available in the .yaml configuration files in the SNAKE source repository.^3^

**Table 2.** Overview of simulated scenarios and their computation requirements—*L*, *N_s_*, *n_jobs_* specify the number of simulated coils, the number of shots acquired to create a k-space volume, and the number of concurrently simulated shots, respectively.

									Time	
Setup	Res.	Readout	$SNR_{i}$	L	$N_{s}$	$T_{obs}$	$TR_{vol}$	$n_{jobs}$	Fourier (6)	With $T_{2}^{*}$ (5)
Scenario 1	3 mm	EPI	1,000	1	44	25 ms	2.2 sec	6	3 min54 sec	3 min55 sec
Scenario 2	3 mm	SoS	1,000	8	14	30 ms	0.7 sec	6	1 min31 sec	5 min10 sec
Scenario 3	1 mm	SPARKLING	30	32	48	25 ms	2.4 sec	3	1 h34 min20 sec	3 h58 min40 sec

*Hardware*: CPU: Intel i9-13900H, RAM: 32Go, GPU: NVIDIA RTX 2000 Ada.

*Common sequences parameters*: *TR_shot_* = 50 ms, *TE* = 25 ms, *FA* = 12°.

As the unitary $TR_{shot}$ and the simulation time (5 min) are the same for all scenarios, each simulation has a budget of 6,000 shots to acquire. Depending on the number of shots allocated per frame (for a specific acceleration factor AF), we end up with a variable number of k-space volumes across the three scenarios.

The simulation times reported in Table 1 underline the additional overhead of using Eq. (5) instead of Eq. (6) for the simulation. In Scenario 1, the simulation time is limited by I/O communications, whereas Scenarios 2 and 3 are computationally bounded.

#### 5.1.2 Scenario S1: 3D fully sampled Cartesian readout

As a first validation example, we simulated the acquisition of 3D Echo Planar Imaging (EPI) data, as an implementation of a 3D Cartesian readout. The 3D EPI is segmented plane by plane in the k-space (as in Poser et al., 2010). Each slice was fully sampled at an isotropic resolution of 3 mm (matrix size: 60×71×60 ), with a $TR_{shot}=50$ ms. The volume-wise temporal resolution is $TR_{vol}=2.2$ sec at Ernst flip angle ($12^{\circ }$), leading to an $SNR_{i}$ of 1,000 calibrated on real acquisitions (cf. Section 3.3). Since the data were collected at the Nyquist rate, we do not need to resort to parallel imaging; hence, we restrict ourselves to a single-coil acquisition. This simple configuration can be simulated on a standard laptop in a few minutes; a similar configuration acquired with POSSUM would have taken several hours, for fewer slices (see Drobnjak et al., 2006; Table 2), due to its model more mathematically involved related to MR physics.

#### 5.1.3 Scenario S2: 3D under-sampled stack of spirals (SoS) readout with VDS acceleration along the stacking axis

**Fig. 3.**
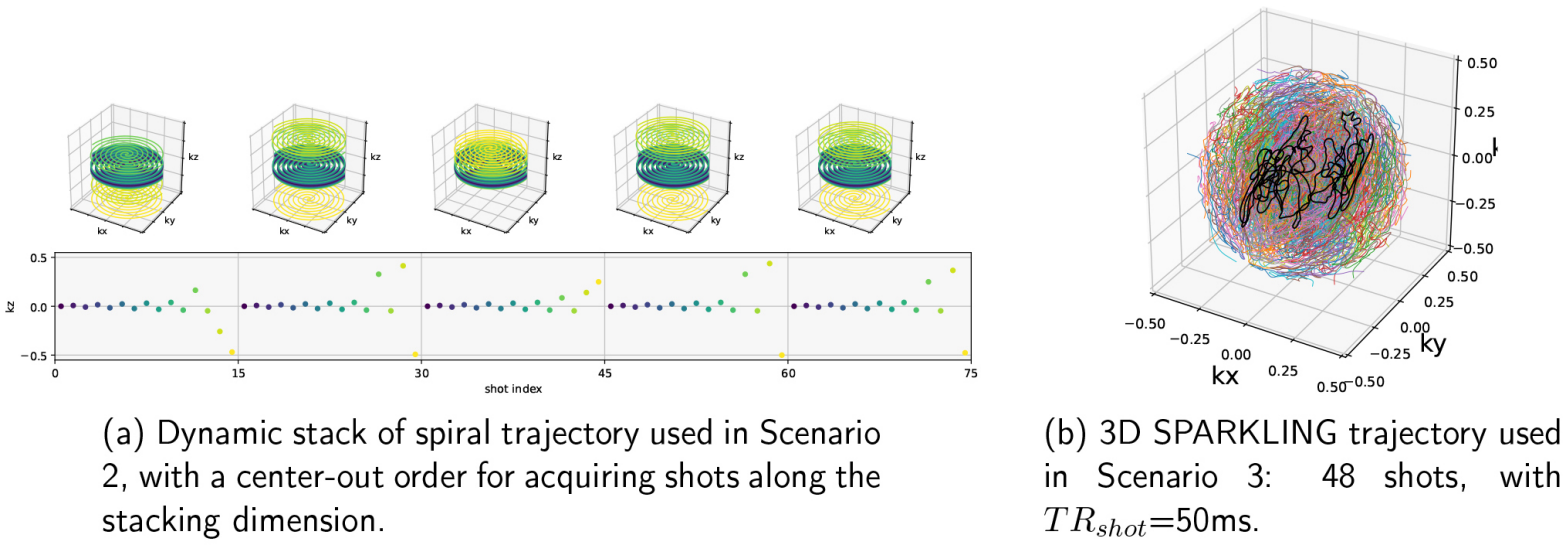
Non-Cartesian k-space sampling trajectories used in Scenarios 2 and 3, respectively.

The second scenario explored the possibility of SNAKE for accelerated imaging based on Compressed Sensing (CS) for data acquisition and image reconstruction. The resolution and FOV remained the same as for Scenario S1, but the goal was to increase the temporal resolution. To do so, we performed an acceleration on the 2D plane ($k_{x},k_{y}$) using an in-out spiral acquisition and second we implemented a spiral stack using a variable density sampling along $k_{z}$, that is, the stacking dimension (see parameters in Table 2). This sampling evolved across scans: Around 10% of the center planes were constantly acquired, while we used an acceleration factor AF = 4 on the outer planes, as shown in Figure 3a. We eventually collected 14 spiral shots per volume, reaching $TR_{vol}=0.7s$ as a temporal volume resolution ($TR_{vol}$ ). To compensate for aliasing artifacts due to undersampling, we simulated multicoil acquisition with L=8 receiver coils. Using GPU-accelerated NUFFT, the simulation time of the acquisition at 3 mm isotropic resolution took around 1.5 min of computation (Table 2). The CPU-based NUFFT implementation is also available, but remains slower.

This approach has already been studied numerically and experimentally in Petrov et al. (2017) with image reconstruction strategies that take advantage of this acceleration mechanism, such as low rank+sparse regularization (Chiew et al., 2018; Lin & Fessler, 2019; Otazo et al., 2015; Petrov et al., 2017). However, these methods reconstruct the complete sequence of image volumes based on the whole 3D+time k-space dataset. Here, we limit image reconstruction strategies to frame-wise approaches, described in Section 5.2.

#### 5.1.4 Scenario S3: Fully 3D SPARKLING under-sampled readout

We finally simulated a third scenario using a fully 3D non-Cartesian sampling pattern, based on 3D SPARKLING acquisition (Amor, Ciuciu, et al., 2024; Chaithya et al., 2022) over L=32 receiver coils at an isotropic resolution of 1 mm and $TR_{vol}=2.4$ sec. 3D SPARKLING implements a variable density sampling according to a prescribed sampling density in the k-space, while complying with the hardware constraints on the magnitude of the gradient $G_{max}$ and the slew rate $S_{max}$ . These values are user-defined and were set to $G_{max}=40$ mT/m and $S_{max}=180$ T/m/s. The target density was a radially decaying distribution parameterized by a cut-off and decay parameters set to (C,D)=(0.25,2); see Amor, Ciuciu, et al. (2024) and Eq. (3) for details. Fully 3D SPARKLING allows us to further accelerate the acquisition process compared to Scenario 2 to reach higher spatial resolution. As we move to higher spatial resolution compared to scenarios S1 and S2 (voxels are 27 × smaller), we divided the level SNRi by a 30-fold factor to mimic the reality of MR physics.

The parameters for this scenario are based on the experimental setup of Amor, Ciuciu, et al. (2024), which is described in Section 5.2, and in Figure 3. In contrast to Scenario 2, here we adopted the “scan and repeat mode” that consists of sampling the same k-space locations across consecutive frames. Time-varying 4D SPARKLING acquisition for fMRI is left for future work and currently beyond the scope of SNAKE.

### 5.2 Reconstruction strategies for Scenario 2 and 3

For simplicity, as the primary focus here was on the simulation of k-space data, we restrict ourselves to a classical, frame-wise CS based image reconstruction with a standard sparsity-enforcing regularization term in the spatial wavelet domain:

$${\hat{x}}_{t}=\text{arg}\underset{x\in {\mathbb{C}}^{M}}{\text{min}}\frac{1}{2}\sum_{\ell =1}^{L}∥{ℱ}_{\Omega_{t}}S_{\ell }x-y_{t,\ell }{∥}_{2}^{2}+\;\mu_{t}g\left(\Psi x\right).$$ (10)

This means that each frame t is reconstructed independently of the others, as we use the following notation:

- ${ℱ}_{\Omega_{t}}$ is the Fourier transform operator for the trajectories grouped in the sampling pattern $\Omega_{t}$ in the frame t.
- Ψ is an orthogonal wavelet transform such as sym-8.
- $\mu_{t}>0$ is the regularization parameter for frame t.
- g(⋅) is the regularization function; here, a standard $\ell_{1}$-norm is used.

The cost function to be minimized is convex but non-smooth. Its global minimizer can be found iteratively using a wide range of proximal gradient methods with possible acceleration schemes such as FISTA, POGM (Beck & Teboulle, 2009; D. Kim & Fessler, 2018). As illustrated in Figure 2, CS-based image reconstruction was performed using the PySAP library (Farrens et al., 2020), and the implementation of the POGM algorithm (D. Kim & Fessler, 2018). Specifically for fMRI, we implemented a dedicated plugin called pysap-fmri. To reduce the number of free parameters, the regularization parameter for each frame $\mu_{t}$ was estimated using Stein’s unbiased risk estimate (SURE) principle (Donoho & Johnstone, 1994), as detailed in Appendix B.

**Fig. 4.**
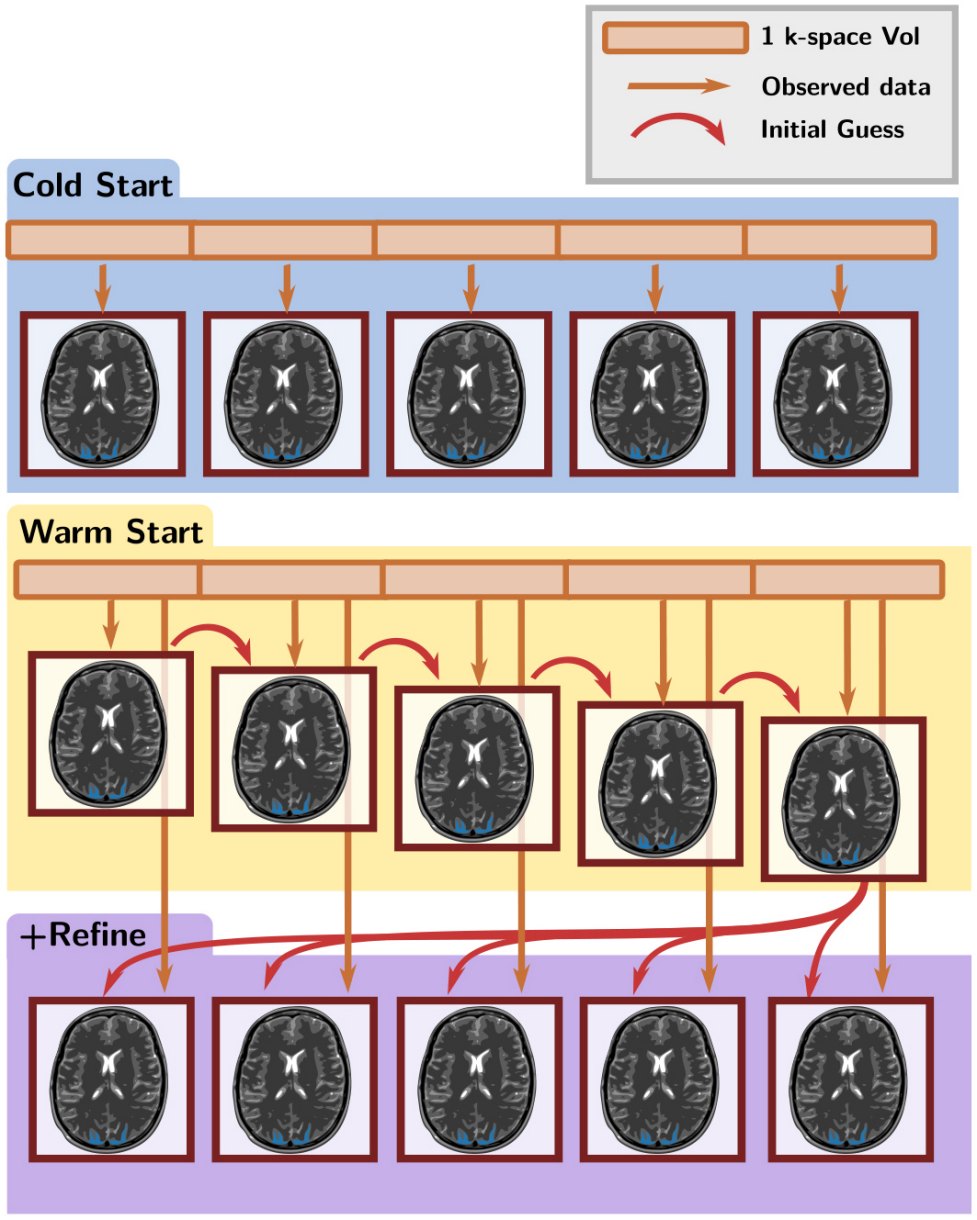
Different methodologies for sequential reconstruction used in Scenario S2. Top: *cold start* reconstruction, each frame is reconstructed independently. Center: *warm start* reconstruction, each frame is reconstructed using the previous frame as initialization. Bottom: *refined* reconstruction, after a warm start reconstruction, the last frame is used as initialization for all other frames.

The versatility of SNAKE allowed us to investigate two different acquisition strategies within scenario S2, the first being based on *static* SoS in k-space whereas the second is associated with a *dynamic* SoS. In the static regime, a constant spiral stack was used for all frames, which means that $\Omega_{t}\;\;=\;\;\Omega ,\forall t$ , whereas in the dynamic regime, a spiral stack $\Omega_{t}$ that varied as a function of the frame t was designed by randomly picking up $k_{z}$ plans for each frame, as shown in Figure 3a. Then, CS reconstruction of each frame, as described in Figure 4, was carried out according to three different mechanisms:

(i) in a *cold-start* manner, where each volume in frame t is reconstructed by solving Eq. (10) independently of previous frames {1,…,t−1}, as illustrated in Figure 4[top].
(ii) in a *warm-start* manner, where the volume reconstructed in frame t by solving Eq. (10) is used as initialization to reconstruct the following volume frame t+1 , as shown in Figure 4[center].
(iii) in a *smart* manner, using an additional *refined* initialization, where the last volume reconstructed in frame T using the previous warm-start strategy was eventually injected as a new set-up for all previous frames, as explained in Figure 4[bottom]. Coupled with dynamic SoS acquisition, this approach is instrumental in visiting all k-space measurements across consecutive frames.

All these variations are based on a frame-wise 3D image reconstruction strategy and, as such, provide a memory-efficient implementation. However, they do not leverage all 4D fMRI k-space data at once, in contrast to low-rank+sparse methods (Otazo et al., 2015; Petrov et al., 2017). This comparison is left for future work.

Estimation of $\mu_{t}$ in each frame (detailed in Appendix B) plays a critical role in the performance of strategies *warm* and *refined*. The first reconstructed frames are highly regularized, but as we progress toward the end of the run, the estimates of $\mu_{t}$ using the SURE-based methods get smaller, as we progressively embed more information to reconstruct volume $x_{t}$.

### 5.3 Evaluation methods

To evaluate the performance of each scenario and the impact of data acquisition and image reconstruction strategies, each combination was submitted to a standard fMRI statistical analysis pipeline, which consists of applying a general linear model (GLM) and testing the positivity of the single modeled experimental condition (visual) in the design matrix. Then, a Student T statistic associated with the regression parameter β was formed and thresholded at p<0.001 (one-sided), uncorrected for multiple comparisons. However, since statistical analysis is deferred to the nilearn package, it is straightforward to introduce a family error rate control if necessary.

As we know the ground-truth activated ROI, we can determine which detected activations are the true/false positives and negatives. The region of interest being small, we have a strongly imbalanced dataset and used the Precision/Recall curve instead of the classical receiver operator characteristic (ROC) curve to accurately compute for each scenario both the area-under-curve (AUC) and balanced accuracy (BACC) scores at p<0.001 . Additionally, we also measured image quality metrics (PSNR and SSIM) as well as temporal SNR (tSNR), to assess the quality of the reconstruction in space and time.

## 6 Results

### 6.1 Scenario S1

**Fig. 5.**
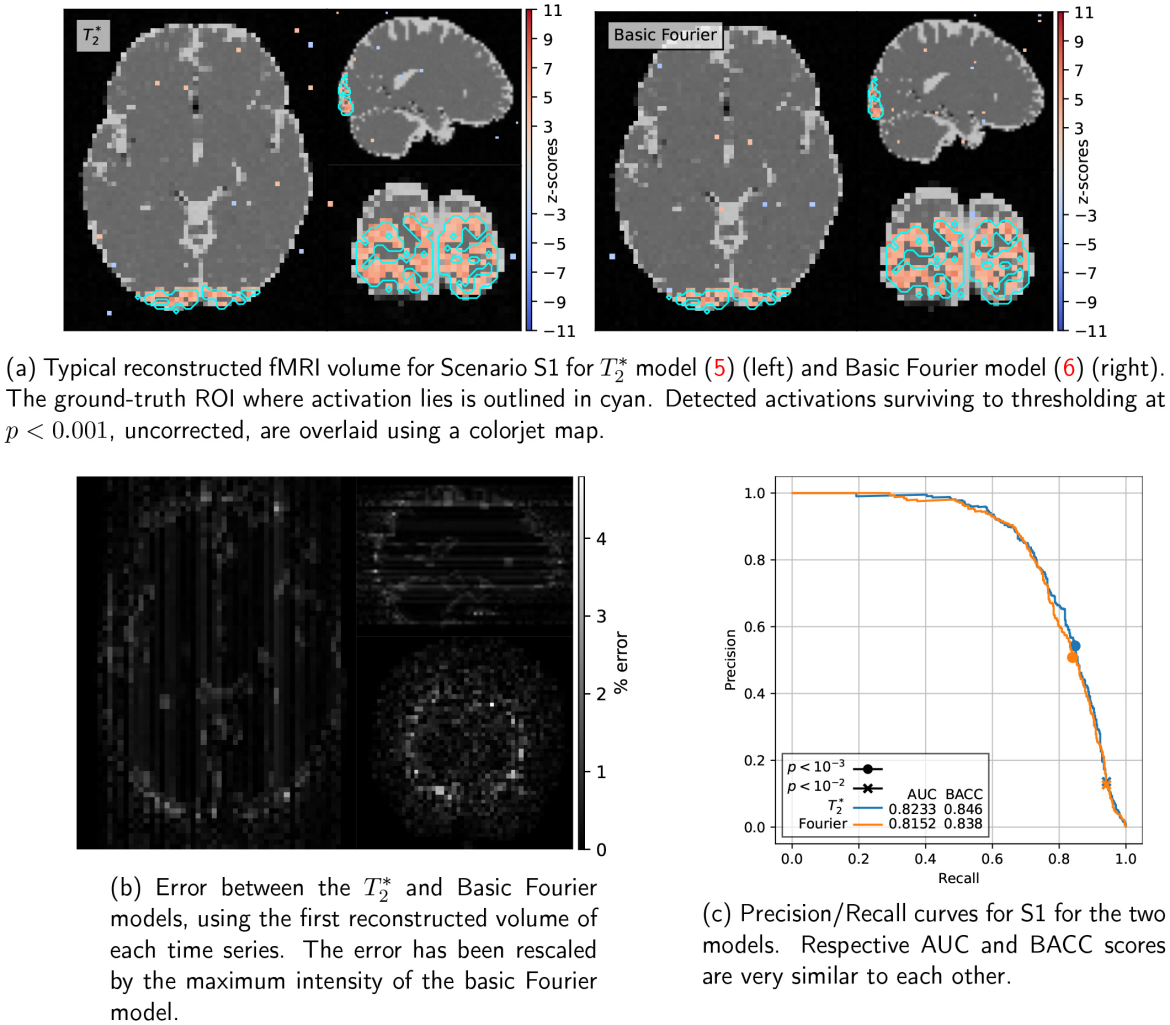
Comparison of the two acquisition models (basic Fourier and $T_{2}^{*}$ effects) for scenario S1.

In Scenario 1, the k-space was fully sampled using EPI planes and reconstructed using the inverse FFT. The low spatial and temporal resolution results in high SNR and good image quality, as shown in Figure 5a. $T_{2}^{*}$ relaxation only introduces negligible artifacts (5% of maximal error, see Fig. 5b), and does not affect statistical performance compared to the basic Fourier model, as illustrated in Figure 5c. In general, this scenario validates the ability of SNAKE to handle end-to-end fMRI simulation and reconstruction.

### 6.2 Scenario S2

Scenario S2 focuses on the joint effect of optimizing acquisition and reconstruction strategies for acceleration purposes and therefore demonstrates the versatility of SNAKE. As described in Section 5.1.3, two acquisition strategies (static vs. dynamic stack of spirals) compete and three variations of reconstruction methods (cold vs. warm vs. refined initialization for each volume) are studied.

**Fig. 6.**
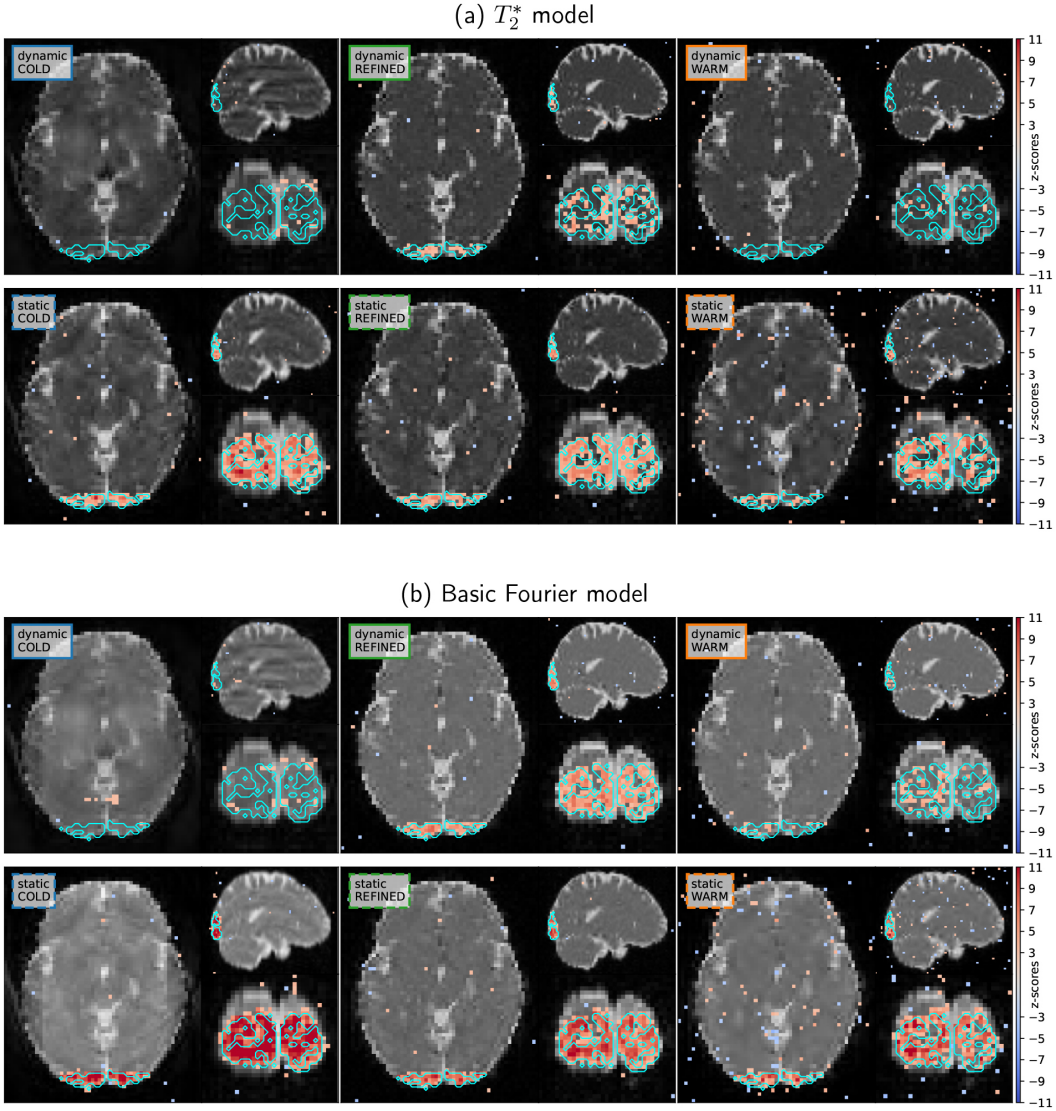
Activation maps for scenario S2. Top: $T_{2}^{*}$ relaxation is taken into account. Bottom: Basic Fourier model, no $T_{2}^{*}$ relaxation. Colored frames within each insert follow the convention adopted in the tables reported in Figure 7. Detected activations surviving to thresholding at p<0.001 , uncorrected, are overlaid using a colorjet map.

**Fig. 7.**
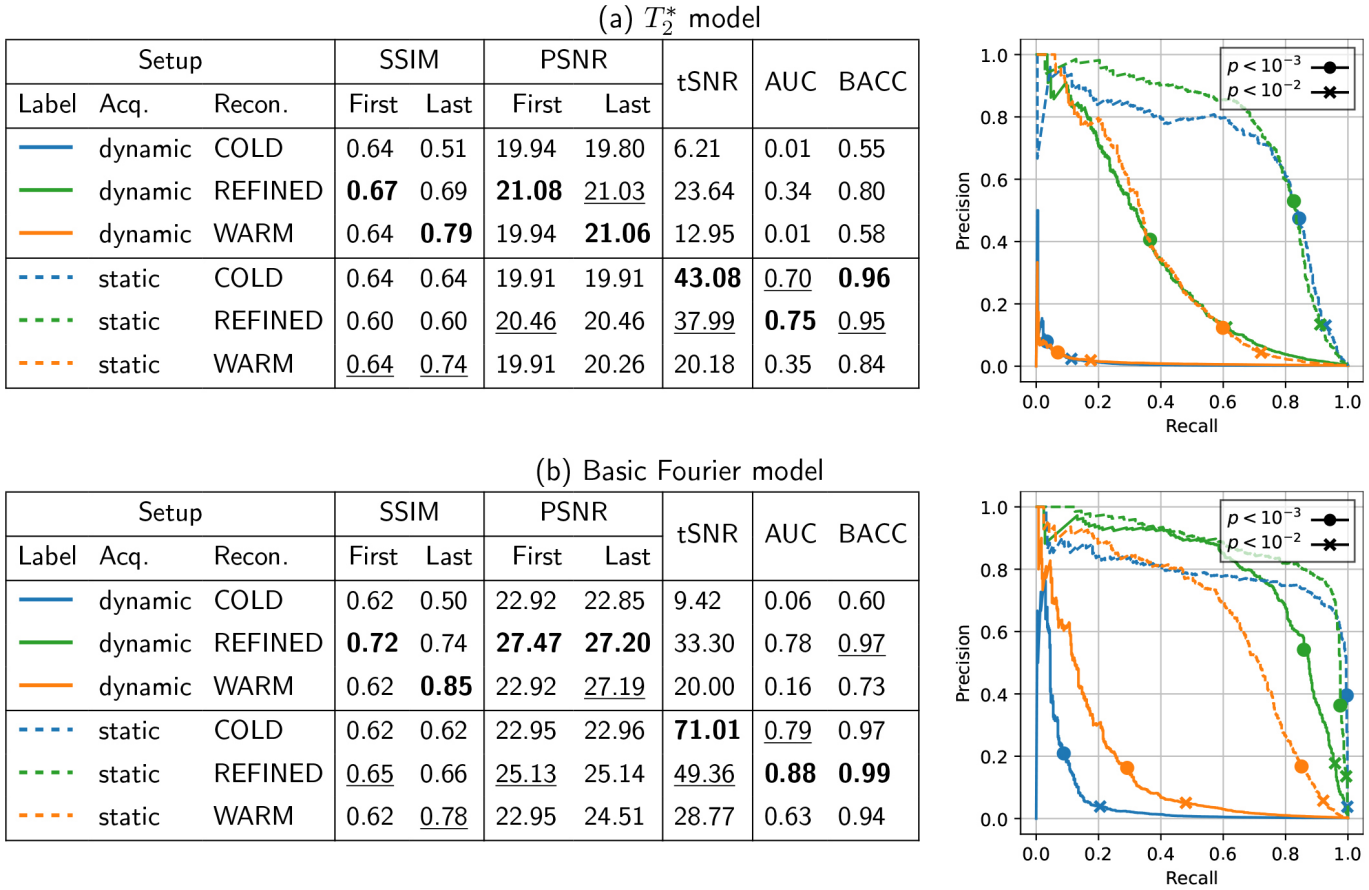
Quantitative metrics summarizing image quality (SSIM/PSNR), signal quality (tSNR), and statistical performances (AUC/BACC) for scenario S2. Top: $T_{2}^{*}$ relaxation is taken into account. Bottom: Basic Fourier model. TSNR values are averaged over the occipital ROI. In the tables, First and Last refer to the first and last frames in the fMRI volume sequences.

The image quality and statistical maps are shown in Figure 6, and the extensive quantitative statistical analysis is reported in Figure 7. Based on these results, we can make the following claims.

#### 6.2.1 $T_{2}^{*}$ relaxation must be taken into account for the long readout in non-Cartesian acquisitions

The $T_{2}^{*}$ relaxation model should not be neglected in the simulation stage when considering non-Cartesian readouts instead of 3D EPI for comparable TE and observation time $T_{obs}$ , as its impact on both image quality and statistical performance is significant. In particular, the comparison of the results shown in Figure 7a and b shows a significant drop in the SSIM, PSNR, and tSNR scores for the relaxation model $T_{2}^{*}$ due to blurring and contrast loss. This decrease in image quality occurs because the reference MR image is the ideal phantom with contrast at TE , which does not suffer from the relaxation of $T_{2}^{*}$. Similarly, we observe a decrease in AUC/BACC for the $T_{2}^{*}$ relaxation model, as well as a loss of precision and recall. This loss in precision is the result of lower z-scores for that model compared to the basic Fourier model, as shown when contrasting Figure 6a and b.

*Hereafter, we only discuss the* $T_{2}^{*}$ *relaxation model for this scenario, as it is the closest to real fMRI acquisitions.*

#### 6.2.2 The refined initialization is key in dynamic acquisition for improved performances

When comparing image reconstruction strategies, we observe in Figure 7 that the warm-start mechanism is beneficial to improve the image quality between the first and last frames for both models. The gain in both the SSIM and the PSNR scores is actually large when this strategy is activated. However, activating only the first warm start strategy does not yield improved statistical performance. Instead, using the *dynamic refined* approach in the reconstruction stage adds value to image quality and statistical performance, notably for the dynamic acquisition setup. In this specific scenario, this reconstruction strategy allows each frame to actually bring new information from complementary undersampled k-space data, and thus maintain good performance while increasing temporal resolution.

Furthermore, the use of time-varying undersampling patterns introduces temporal incoherence between frames, and thus introduces different aliasing artifacts for each frame, which can be detrimental to image quality and statistical performances (see for instance, the large gaps in the different scores between the strategies *dynamic cold* and *refined* in Fig. 7).

#### 6.2.3 Image quality is not the right proxy for good statistical performances

The static acquisition strategy provides the best statistical results, as it allows us to detect evoked brain activity in the targeted ROI with the highest precision/recall and tSNR scores (cf. Fig. 7a). Here, the tSNR metric correlates well with AUC/BACC scores as the noise is purely thermal (no physiological noise). However, image quality is degraded in these cases; this is notably visible in Figure 6 when comparing the results *static cold* to *dynamic refined*. Strong aliasing and blurring artifacts actually corrupt reconstructed MR images in the static regime. Additionally, consistency of image quality (even in the degraded case) over the whole fMRI run also matters, as warm-start reconstruction (where image quality improves over time) shows poor statistical performance.

Finding the best trade-off between image quality and statistical performance can be achieved using the refined mechanism in terms of initialization strategy, as it provides the best image quality throughout the sequence of fMRI volumes while retaining good statistical performance. This is particularly noticeable in the dynamic acquisition setting: The AUC/BACC scores associated with the refined strategy (solid green curve) are higher than those associated with warm/cold strategies (solid blue and orange curves); see Figure 7a for the $T_{2}^{*}$ model. More strikingly, this also happens in the static acquisition setting. A significant gain in SSIM/PSNR is observed with the refined strategy, and still the AUC/BACC scores are comparable to those measured using the cold initialization. When comparing the green and blue Precision / Recall dashed curves in Figure 7a, one can even observe that the green bullet is on top of the blue one, indicating a slight boost in precision (statistical sensitivity) at a given specificity (i.e., recall) level (same value along the x-axis). Finally, all these observations that were established on the $T_{2}^{*}$ model remain valid in the simplified Fourier model (cf. Fig. 7b), which enforces these statements.

However, dynamic acquisition strategies have shown great potential to increase temporal resolution while maintaining good image quality. Preserving fine details in fMRI images at the output of the reconstruction pipeline matters for the correct preprocessing of fMRI volumes and reliable statistical analysis (registration to a template, extraction of the cortical surface, etc.).

### 6.3 Scenario S3

Finally, we present the results obtained for Scenario S3 (1 mm isotropic resolution, $TR_{vol}$ = 2.4 sec) as a proof of concept of the scalability of SNAKE to reach high spatial resolution fMRI using 3D non-Cartesian readout such as 3D SPARKLING.

**Fig. 8.**
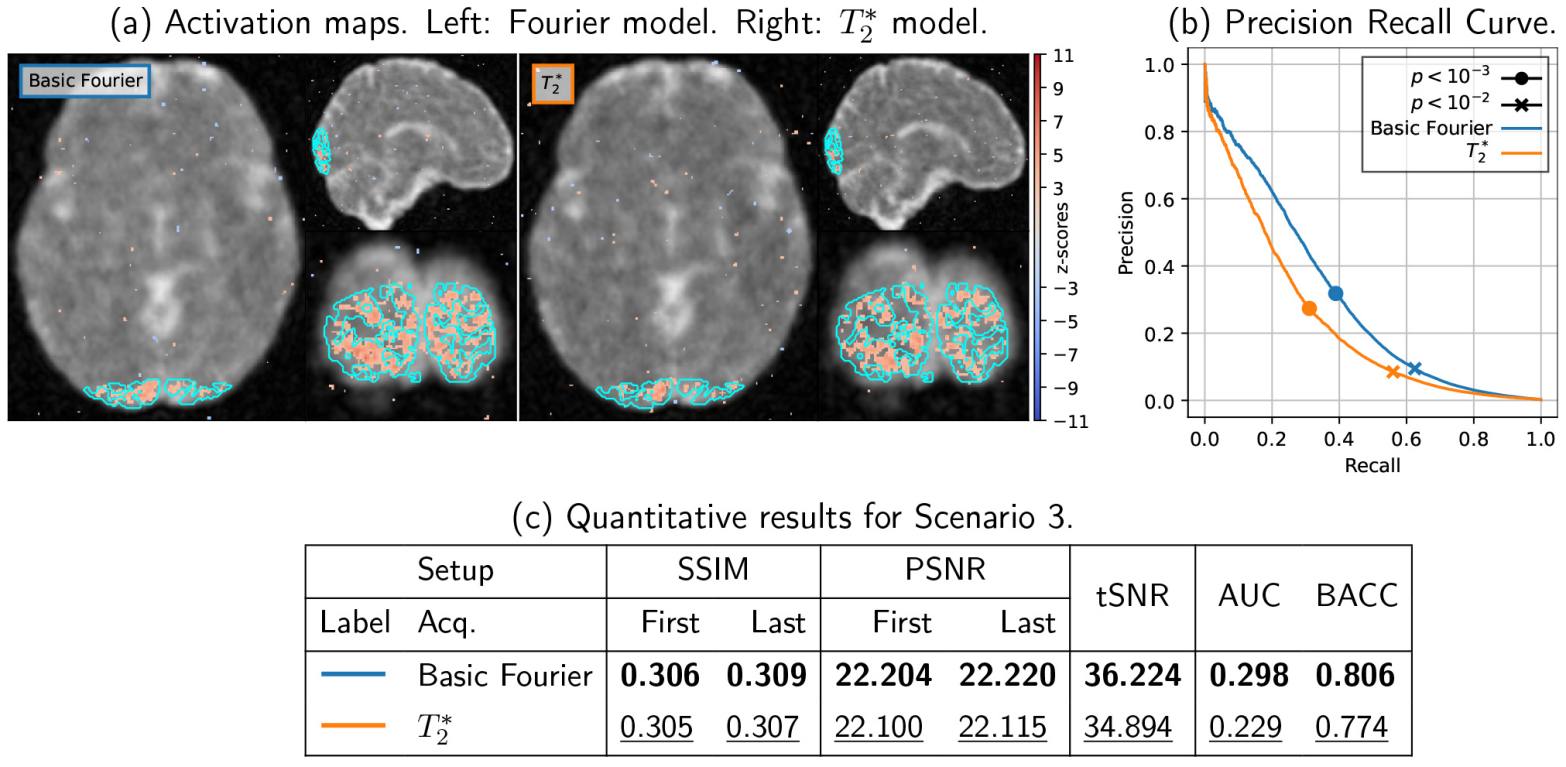
Results for scenario S3. Top left/center: First fMRI reconstructed volume using the standard Fourier (left) and $T_{2}^{*}$ (center) models. Sequential cold-start CS reconstruction was performed frame-wise. Detected activations surviving thresholding at p<0.001 , uncorrected, are overlaid using a colorjet map. Top right: Precision/Recall curves for the two models. Accurate modeling of realistic $T_{2}^{*}$ decay slightly impacts statistical performances. Bottom: Quantitative metrics summarizing image quality (SSIM/PSNR), signal quality (tSNR) and statistical performances (AUC/BACC).

With full GPU acceleration, sequential cold start reconstruction took 4 min4 sec per frame to converge, compared to 15–20 min in the previous implementation (Amor, Ciuciu, et al., 2024), thus requiring 8 h30 min for a complete fMRI run (125 frames). Both models (with and without $T_{2}^{*}$ relaxation) show similar and relatively poor image quality due to blurring, as shown in Figure 8a. Additionally, the statistical performances are slightly lower (loss in sensitivity and specificity) when the $T_{2}^{*}$ relaxation effect is considered in the data simulation process, as illustrated in Figure 8c.

## 7 Discussion

### 7.1 Effect of $T_{2}^{*}$ relaxation

All scenarios have roughly the same $T_{2}^{*}$, TE and $T_{obs}$ , but the $T_{2}^{*}$ relaxation effect depends on the acquisition strategy and the k-space trajectory used. In Scenario 1 (EPI trajectory), no noticeable changes are visible, as the k-space is fully sampled and nearby k-space points are acquired at close times. In Scenario 2, the spiral trajectory allows for the acquisition of two neighboring points in k-space at both temporal extremities of the spiral. This results in a change of imaging contrast between two neighboring measurements in the k-space, thus inducing a loss of contrast in the reconstructed fMRI images. This effect can be alleviated using temporally smooth trajectories such as cones or MORE-SPARKLING (Giliyar Radhakrishna et al., 2023) readouts. Statistical performance is also affected as lower z-scores were retrieved for the $T_{2}^{*}$ model compared to the basic Fourier model, as shown in Figure 6. However, for well-chosen reconstruction scenarios (e.g., static cold/refined), evoked brain activity is still well detected in the occipital ROI, and the precision-recall at p<0.001 remains similar. Similar findings were observed in the case of dynamic acquisition with refined reconstruction.

In general, we recommend keeping the modeling of the $T_{2}^{*}$ relaxation effects in the simulation despite the higher computing cost, as they can still influence the extraction of z-scores as a function of the readout k-space for given TE and $T_{obs}$ .

### 7.2 Setting the noise level and tSNR

**Appendix Fig. A2.**
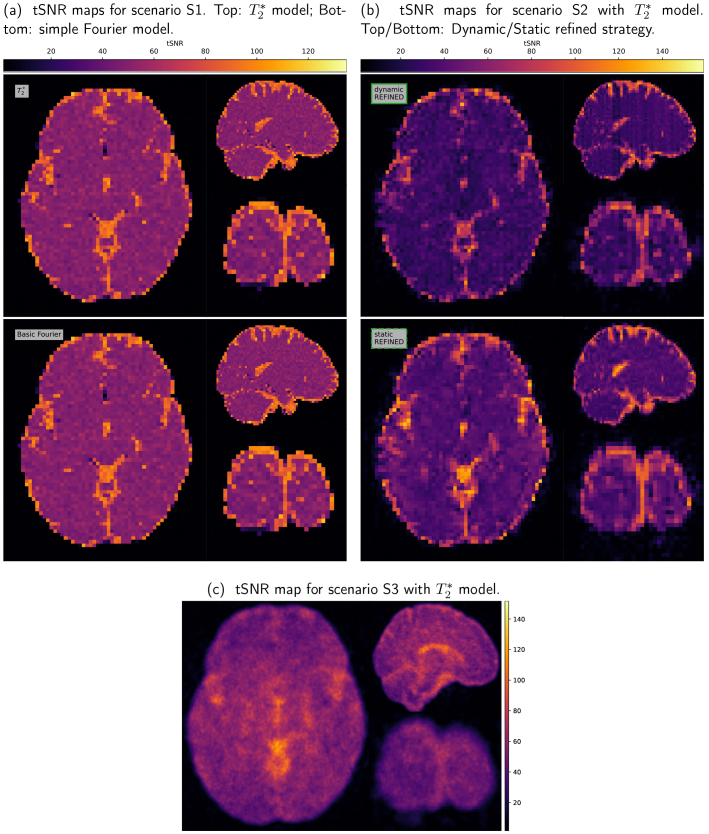
tSNR maps for the 3 scenarios.

Concerning the choice of input SNR, $SNR_{i}$, in Appendix C we reported the tSNR maps for each scenario. In S1, we measured a tSNR of 40.5 for the basic Fourier and $T_{2}^{*}$ models in the occipital ROI. The tSNR increased to 120 in the CSF, as shown in Appendix Figure A2a. This is in line with what we could expect under experimental conditions. TSNR values for S2 are reported in Figure 7. As the $T_{2}^{*}$ relaxation is significant in this scenario, we can see a decrease in tSNR, which is accompanied by lower statistical performances. However, tSNR is not an oracle for statistical performance, as outlined in Jamil et al. (2021). Finally, in S3, we get an tSNR of 36 in the occipital ROI. By decreasing SNRi at higher resolution from 1,000 at 3 mm iso to 30 at 1 mm iso, we obtained similar tSNR ranges in the three scenarios. Note that for S3, similar values have been empirically observed in Amor et al. (2022).

### 7.3 Insights from the different scenarios

The three scenarios presented in Sections 5 and 6 provide complementary views on the comparison of acquisition and reconstruction strategies.

Scenario S1 covers a low spatial and temporal resolution setting, uses standard Cartesian acquisition and basic reconstruction methods, and shows the potential of SNAKE to produce a basic reference for detecting evoked brain activity at a given input SNR. Beyond providing a simple and fast validation case for the simulator, this scenario could be used to compare the statistical performances of different competing experimental paradigms regarding the number and duration of stimuli, block versus event-related designs, for a given scan time budget, and under various artifacts.

Scenario S2 explores a low spatial, high temporal resolution setup and provides a large panorama of possible acquisition and reconstruction strategies. It shows the potential of SNAKE to produce a benchmark of competing techniques both at acquisition (static vs. dynamic stack of spirals) and image reconstruction stages, particularly in the compressed sensing framework. This benchmark could be extended in the near future using more complex methods such as low-rank $\tilde{+}$ sparse methods or even deep learning approaches such as unrolled techniques (Ramzi et al., 2022; Tanabene et al., 2024) or plug-and-play methods (Comby, Lapostolle, et al., 2025; Comby, Terris, et al., 2025). Interestingly, from its current implementation, we observed that the best image quality is not always associated with the highest statistical scores, and that the refined initialization strategy is key in dynamic acquisition for improved statistical performances.

Finally, Scenario S3 provides a high spatial and low temporal resolution setup, reaching the limits of current fMRI acquisition strategies for whole-brain coverage. Unlike S2, the SPARKLING under-sampling pattern does not show aliasing artifacts, even at high spatial resolution, however, at the cost of some blurring. More generally, SNAKE offers new possibilities to optimize further 3D and even 4D under-sampling patterns in the near future.

In general, these three scenarios should be considered as an upper bound in terms of image quality and statistical performances for prospective validation on real fMRI experiments, as actual fMRI data acquisition and image reconstruction face additional issues such as imperfection in coil sensitivity estimation, presence of motion artifacts, and off-resonance effects due to static and dynamic $B_{0}$ inhomogeneities.

### 7.4 Limits to the study

Our work is well defined by the hypotheses formulated in Section 3.4, and their practical application in Section 5. In particular, the scenarios presented in this paper omit three major sources of noise in the fMRI data: head movement, physiological effects, and inhomogeneities of $B_{0}$, since we focus on the core capabilities of SNAKE. Similarly, we only used the three main tissue classes (WM, GM, CSF) to accelerate the simulation of the fMRI data in the k-space. This choice was also the consequence of missing information in the literature on relaxation parameter values for other tissue classes at 7T. Moreover, the Cartesian scenario restricts itself to fully sampled EPI and lacks any acceleration setup such as GRAPPA or CAIPI (Breuer et al., 2005; Griswold et al., 2002). Future work will address these aspects notably using the recently developed generalized GRAPPA package (Bertrait et al., 2025) and explore more challenging reconstruction setups. However, SNAKE provides neuroscientists with an upper limit on the statistical impact and performance of many combinations of acquisition and reconstruction strategies, which offers an easy exploration of new methodologies.

#### 7.4.1 Why off-resonance effects are not considered in this study

At first sight, the off-resonance effects due to static and dynamic $B_{0}$ inhomogeneities could be considered as a major source of artifacts in fMRI, especially for non-Cartesian acquisition strategies. However, even in the absence of their simulation, we can already see that it is possible to discriminate acquisition and reconstruction strategies regarding their statistical performances in the downstream analysis. Furthermore, in real-life acquisition, various methods are put in place to mitigate these effects (e.g., passive and active shimming, eddy current compensation, field monitoring, etc.). *From this standpoint, the generated data using SNAKE could be considered a best-case scenario, as it is already possible to reach in experimental settings*. We are comforted by the comparable image quality obtained by SNAKE (if not slightly worse) and experimental fMRI data, for example, in scenario 3 which follows the experimental setup of Amor, Ciuciu, et al. (2024), where the field camera was used to correct for static and dynamic off-resonance effects, as well as first-order errors in the gradient waveforms (i.e., in k-space trajectories). Finally, our experiments simulate activation in the primary visual cortex, which stays away from areas of the brain (such as the ear canals and sinuses), where off-resonance effects are typically observed. Therefore, the simulated scenarios that have been proposed can be regarded as reliable.

On the more practical side, faithful modeling of the off-resonance effects would require a dramatic increase in computational requirements, which would be detrimental to the ease of using SNAKE and the speed of the development cycle of new acquisition strategies. Modeling accurate off-resonance effects would require: *(i)* A high-resolution static field inhomogeneity map: To obtain such a map, it would be needed to increase the acquisition resolution of the simulation (e.g., from 3 to 0.5 mm^3^ and the number of tissues considered (from $N_{tis}=3$ to $N_{tis}=12$ in Eq. (5))), as they are all required to obtain a correct model of the air-tissue interfaces of the head. *(ii)* Applying such a high-resolution field map with high fidelity using Eq. (5) would also increase the number of interpolators to P=100 .

Thus, the number of Fourier operator calls being $N_{tis}\cdot P\cdot L$ , it would lead to 1,200 calls to high-resolution NUFFT operators instead of 400 calls at lower resolution, and very high memory requirements with arrays of size $N_{tis}\times N_{voxels}$ for the phantom, $L\times N_{voxels}$ coil-sensitivity maps, as well as $P\times N_{samples}$ and $P\times N_{voxels}$ for the off-resonance maps (resulting in ≃70GB of GPU memory required per shot). In comparison, our scenario 3 with 1 mm^3^ resolution looks far easier to compute.

Finally, without considering oracle knowledge of the field map (In practice, a separate acquisition is required, which may be limited in precision), image reconstruction at the target 3 mm iso will also be a challenge: The interpolator model will also have to be used for reconstruction. In general, this would severely hinder the scalability of SNAKE and its ability to explore new strategies for both acquisition and reconstruction.

#### 7.4.2 Restricting to single-echo GRE fMRI

In this study, we focused on single-echo GRE fMRI, which is the most common sequence used in fMRI experiments, in particular at an ultra-high field. The aim of SNAKE is, in the first place, to push the boundary of what is possible in terms of spatial and temporal resolution for whole-brain acquisition with ultra-high field MRI. Thus, even if the modularity of the SNAKE acquisition engine would allow it, we did not consider the multi-echo GRE fMRI setup (a crude solution would be to run SNAKE for each echo and then combine the results, while ensuring that the overall parameterization is consistent across echoes and realistic). Furthermore, multi-echo setups are typically reduced in terms of spatial resolution at ultra-high magnetic field, as multiple scans of the k-space need to be performed during a shorter $T_{2}^{*}$ decay.

Similarly, we did not consider spin-echo-based sequences; they are not as common as GRE in fMRI, and the extra acquisition time and SAR constraints make them less attractive for ultra-high field fMRI. However, the modularity of the SNAKE acquisition engine would allow it to simulate such sequences, by replacing the GRE steady-state signal model (2) with the appropriate spin-echo signal model.

### 7.5 Extending the simulator

As SNAKE is open-source software, external contributors from the fMRI community are welcome to participate in its extension to help refine the forward model of fMRI data which could take into account multiple sources of artifacts. In addition to head motion and off-resonance (of which we are adding some preliminary support), we may think of modeling temporal aliasing artifacts due to physiological rhythms (heart beat or breathing rate) that are not sampled fast enough. Adequate fMRI acquisition and reconstruction methods could be studied with SNAKE to mitigate these additional sources of disturbance.

Similarly, more complex brain activation patterns spread over multiple ROIs (e.g., using functional atlases from Yeo et al., 2011) could be designed with spatial variations in HRF shape, following the seminal work of PyHRF (Vincent et al., 2014), or using meta-analyses and Python tools such as neurosynth^**^ (Yarkoni et al., 2011) or Neuroquery^††^ (Dockès et al., 2020) to define well-localized activation peaks for given cognitive paradigms and tasks. Moreover, the BOLD effect could also be modeled to have an impact on the phase of the complex-valued signal (S.-G. Kim & Ogawa, 2012; Menon et al., 1992). As the vast majority of fMRI studies focus on the magnitude of the signal, we did not consider these effects in this version of SNAKE.

So far, SNAKE has focused on brain mapping tasks in task-related fMRI. However, it could address the simulation and analysis of resting-state fMRI and be tuned to optimize the retrieval of resting-state functional networks from synthetic rs-fMRI datasets. In that regard, coupling with other fMRI simulators, such as Virtual Brain (Schirner et al., 2022), could be instrumental in generating realistic rs-fMRI data as input reference data to SNAKE. As SNAKE is already interfaced with the nilearn package for statistical analysis and because the latter allows for functional connectivity analysis, this extension to the synthesis and analysis of rs-fMRI data could be quite straightforward.

However, the complexity of the simulation should be balanced with the need for interpretability of the results. On the one hand, adding layers (and their potential heavy parameterization) to the simulation will also induce a potential loss in explicability for the effect on downstream applications. On the other hand, the modularity of SNAKE allows ablation studies to enable/disable any aspect of the simulation and quantify its impact.

### 7.6 Exploring the effect of tuning acquisition and reconstruction together

The first results obtained with SNAKE show the criticality of matching and tuning the experimental design, acquisition, and reconstruction strategies to obtain the best quality in the subsequent statistical analysis. More complex acquisition and reconstruction methods could leverage the temporal redundancy in k-space data with global *a priori* such as low rank+sparse (Graedel et al., 2022; Petrov et al., 2017). The *refined* strategy introduced in Scenario S2 shows some great potential for both static and dynamic acquisition schemes, and will be at the core of future development.

To our knowledge, as summarized in Table 1, SNAKE is the only open-source fMRI data simulator that can efficiently provide arbitrary fMRI k-space data. It is also the only one that can be used to benchmark reconstruction methods in an automated manner.

### 7.7 More than an fMRI simulation tool

The modular approach of SNAKE also enables applications other than simple simulations. First, the combination of the acquisition, reconstruction, and analysis modules provides a reliable benchmark for image reconstruction methods (even for a single anatomical volume).

Second, each handler can also be viewed (and used) as a data augmentation layer for supervised deep learning methods, opening up new opportunities for fMRI image reconstruction. Currently, such models cannot be trained in the supervised setting due to the lack of ground-truth (i.e., non-accelerated) real high-resolution fMRI data. Alternative self-supervised approaches based on domain undersampling (Demirel et al., 2021) are limited in terms of performance, notably in high-resolution settings. Hence, SNAKE could be used to train supervised deep neural networks dedicated to fMRI image reconstruction on synthetic fMRI data. These models might be fine-tuned later with transfer learning on real datasets. Alternatively, SNAKE might serve as a data enhancement tool in real fMRI data. The recent review by Gopinath et al. (2024) outlined the need for efficient synthetic data generation, a task for which SNAKE was precisely designed.

## 8 Conclusion

In this paper, we have proposed a new fMRI data simulator, called SNAKE, which is packaged as an open-source Python software, offering the ISMRM and OHBM communities the opportunity to advance the field of optimal fMRI data acquisition and image reconstruction at low scanning cost. More specifically, SNAKE is purposely designed to assess the impact of massively undersampled 3D (and 2D) non-Cartesian readouts aiming to reach unprecedented spatial or temporal resolution while maintaining whole-brain coverage, good image quality, and the ability to detect a tiny BOLD effect through statistical guarantees. Through its modular design, SNAKE has been thought to remain open to external contributions as there is room to model additional aspects, notably external sources of artifacts (static and dynamic $B_{0}$ inhomogeneities, motion, etc.).

SNAKE also comes with an extensible and end-to-end reconstruction and statistical analysis pipeline. It provides the user with tools to reach new frontiers in fMRI data acquisition and image reconstruction strategies and to evaluate multiple competing scenarios from an image quality and statistical analysis viewpoint that cannot easily be ranked in advance. Future work will be dedicated to the interface SNAKE with deep learning for fMRI image reconstruction.

## Data Availability

The SNAKE package, its documentation, and the scenarios presented are available at https://github.com/mind-inria/snake-fmri.

## Footnotes

*
https://mind-inria.github.io/snake-fmri

†
https://github.com/paquiteau/pysap-fmri

§ The detail configuration and run scripts for the scenarios of Section 5 are available in https://github.com/mind-inria/snake-fmri.

** https://neurosynth.org/.

††
https://neuroquery.org/

## Appendix B: Regularizer Estimation Using Sure

In Section 5.2, we explained that the regularization parameter $\mu_{t}$ used in Equation (10) was estimated for each frame t. In practice, this is achieved as described in Algorithm 1. As the estimate $x_{t}$ becomes cleaner, the threshold automatically decreases. This estimation is very fast to compute because we restrict the estimation to the most detailed wavelet coefficients in the high-frequency subbands. Therefore, the problem dimension is reduced and we can use a line search strategy to find ${\hat{\mu }}_{t}$ without noticeable overhead for image reconstruction.

**Table:**

**Algorithm 1:** Estimation of $\mu_{t}$ for solving (10)
**Data:** $x_{t},\Psi$ **Result:** $\mu_{t}$ $\alpha_{H}\leftarrow P^{HHH}(\Psi (x_{t}))$ ** **// Extract high-details wavelet coeffs $n\leftarrow \text{size}(\alpha_{H})$ $\sigma \leftarrow \text{MAD}(\alpha_{H})*0.675$ ** **// Estimate variance using Median Absolute Deviation $\alpha_{H}\leftarrow \alpha_{H}\Omega /\Omega \sigma$ **if** $\frac{1}{n}\alpha_{H}^{T}\alpha_{H}<{\text{log}}_{2}{\left(n\right)}^{3/2}\Omega /\Omega \sqrt{n}\;\;$ **then** **$\mu_{t}\leftarrow \sqrt{2{\text{log}}_{2}\left(n\right)}$** **else** **${\hat{\mu }}_{t}\leftarrow \text{arg}\underset{w\in \alpha_{H}}{\min}\sum_{i=1}^{n}\text{min}(\alpha_{H,i}^{2},w^{2})-2*I\{\alpha_{H,i}^{2}<w^{2}\}\;\;\;\;\;\;\;$ **// Find a threshold that minimizes the SURE estimator $\mu_{t}\leftarrow \text{min}\left({\hat{\mu }}_{t},\sqrt{2{\text{log}}_{2}\left(n\right)}\right)$ **end**

## Appendix C: Temporal Snr Maps for the Three Scenarios

To ensure that the values for $SNR_{i}$ have been correctly chosen, here we report the tSNR maps for each scenario. The tSNR in absence of evoked brain activity is defined voxel-wise, in image domain, as:

$$\forall v\in \text{FOV},\;\;\;\;tSNR\left(v\right)=\text{mean}(x_{t}\left(v\right))\Omega /\Omega \text{std}(x_{t}\left(v\right)),$$
(A1)

where $x_{t}(v)$ refers to the BOLD fMRI time series at frame t and location v.

Interestingly, in Appendix Figure A2a we show that the tSNR is marginally impacted by $T_{2}^{*}$ relaxation effects in Scenario 1 due to the use of Cartesian 3D EPI. In regards to Scenario 2, we already reported that $T_{2}^{*}$ relaxation effects were detrimental to the mean tSNR, mainly due to longer $T_{obs}$ . This is confirmed in Appendix Figure A2b with lower tSNR values compared to those shown in Appendix Figure A2a while both scenarios were initialized with the same input SNR and defined at the same spatial resolution. Furthermore, one can observe that the static refined strategy maintains a better tSNR than the dynamic refined one, but is associated instead with a more degraded image quality (significant blurring). Appendix Figure A2c depicts the tSNR map for Scenario 3 under the $T_{2}^{*}$ model, which lies in the same range as for S2 while being smoother spatially.
